# Factors influencing use of telegenetic counseling: perceptions of health care professionals in Sweden

**DOI:** 10.1007/s12687-018-00404-5

**Published:** 2019-01-08

**Authors:** Rebecka Pestoff, Peter Johansson, Per Nilsen, Cecilia Gunnarsson

**Affiliations:** 1 Centre for Rare Diseases in Southeast Region of Sweden, Linköping University, 58185 Linköping, Sweden; 2 Division of Community Medicine, Department of Medical and Health Sciences, Faculty of Medicine Health Sciences, Linköping University, Linköping, Sweden; 3 Department of Genetics, Linköping University, Linköping, Sweden; 4 Department of Social and Welfare Studies, Linköping University, Norrköping, Sweden; 5 Department of Internal Medicine and Department of Medical and Health Sciences, Linköping University, Norrköping, Sweden; 6 Department of Clinical and Experimental Science, Linköping University, Linköping, Sweden

**Keywords:** Genetic counseling, Telegenetic, Implementation

## Abstract

Genetic counseling services are increasing in demand and limited in access due to barriers such as lack of professional genetic counselors, vast geographic distances, and physical hurdles. This research focuses on an alternative mode of delivery for genetic counseling in Sweden, in order to overcome some of the mentioned barriers. The aim of this study is to identify factors that influence the implementation and use of telegenetic counseling in clinical practice, according to health care professionals in Southeast Sweden. Telegenetic counseling refers to the use of video-conferencing as a means to provide genetic counseling. Qualitative, semi-structured interviews with 16 genetic counseling providers took place and phenomenographic analysis was applied. Significant excerpts were identified in each transcript, which led to sub-categories that constructed the main findings. Three categories emerged from the data: (1) requirements for optimal use, (2) impact on clinical practice, and (3) patient benefits. Each category consists of two or three sub-categories, in total seven sub-categories. These findings could potentially be used to improve access and uptake of telegenetic counseling in Sweden and in other countries with a similar health care system. This could benefit not only remote patient populations, as described in previous research, but also large family groups and patients experiencing obstacles in accessing genetic counseling, such as those with a psychiatric illness or time constraints, and be a useful way to make genetic counseling available in the new era of genomics.

## Introduction

Clinical genetics services are struggling to meet increasing demands, partly due to increased awareness about genetic diseases, increased availability of genetic testing and mainstreaming of genetic testing into more general settings due to the genomics era (1K Genome Project 2018). Genetic testing will become more common in general practice; however, according to several organizations, it should still be accompanied by genetic counseling and informed consent (EuroGentest 2010; Organization of Economic and Cooperative Development 2007; The Swedish Government Offices 2006). This is because genetic test results can have an impact on many aspects of a person’s life, such as psychological, medical, social, and practical. It is the responsibility of the genetic counselor to ensure that the patient is appropriately equipped to manage this (Decruyenaere et al. 2003; Skirton et al. 2013). However, the quality of genetic counseling is believed to vary in Sweden due to the varied backgrounds among providers (Pestoff et al. 2016). Further, demand for genetic counseling exceeds the supply of professional genetic counselors contributing to unequal access for patients in some areas or countries.

Several other health care specialties experience a comparable increase in demand, which has prompted a greater use of e-Health (Sveriges kommuner och landsting 2016) and is defined as “using information and communication technologies for health” (World Health Organization 2017). e-Health includes telemedicine that is, “the remote diagnosis and treatment of patients by means of telecommunications technology” (2012) and telemedicine has already been introduced as an alternative in several clinical specialties, such as dermatology (Eissing et al. 2017), ophthalmology (Kim and Driver 2015), cardiology (Lundgren et al. 2016) and in general practice (MinDoktor 2018; KRY 2018; Lorentzon 2018). Telemedicine is particularly appropriate when verbal communication is more important than physical examinations (Hilgart et al. 2012), thus making it suitable for genetic counseling. However, the use of telemedicine in genetics, that is *telegenetics*, is not widely adopted despite being clinically available since 1989. According to a review in 2012 (Hilgart et al. 2012) and one in 2017 (Vrecar et al. 2017), a limited number of pilot studies have been performed on telegenetic counseling (TGC) in Australia, the Netherlands, the UK and the USA. The outcome measures for both patients and HCPs have been largely positive, including among other things: overall satisfaction (Zilliacus et al. 2009; Voils et al. 2017), feasibility (Meropol et al. 2011), outcome equivalent to traditional counseling (Buchanan et al. 2016), increased flexibility (Otten et al. 2016c) and financial aspects (Weissman et al. 2017).

However, most of these studies were limited in scope, primarily investigating TGC carried out at local health care facilities, requiring patients to travel. These studies also focused on evaluating outcomes measures, as mentioned above. There are no, to us known, studies that address factors that need to be in place for use and implementation of TGC according to end users, specifically health care professionals (HCPs) and patients. Factors perceived as negative for the use of TGC are referred to as barriers and factors enabling the use of TGC are referred to as facilitators. In order to achieve successful implementation of innovations in health care settings certain determinants have been identified, for example, the needs, preferences, and experiences of end users (Greenhalgh et al. 2005; Nutley et al. 2007; Damschroder et al. 2009; Nilsen 2015). As suggested by Voils et al. (2017), studies on TGC should not be restricted to provision from a remote health care facility, but instead from the patients’ preferred location, be it at home, at work, or elsewhere. Previously published outcome measures of TGC for both patients and HCPs have measured among other things overall satisfaction, feasibility, financial aspectsand perceived personal control (Abrams and Geier 2006; Stalker et al. 2006; Zilliacus et al. 2009; Zilliacus et al. 2010; Meropol et al. 2011; Otten et al. 2016b, c; Weissman et al. 2017; Voils et al. 2017). Outcomes have ranged from acceptable to positive; however, HCPs have generally been less positive overall, compared to patients (Otten et al. 2016b). The reason for this discrepancy has not been previously explored, nor the factors (determinants) perceived as important in the implementation of TGC identified. Hence, there is a need to understand the perceptions and attitudes of HCPs to TGC in clinical practice. The aim of this study is to identify the factors that influence the implementation and use of TGC in clinical practice, according to HCPs in the southeast region of Sweden.

## Methods

### Study design

This qualitative, inductive study explored the different perceptions among HCPs of hypothetically using TGC in clinical practice. It may be considered prospective as TGC is not yet in use clinically. A phenomenographic approach was applied in order to explore and describe the variations in HCPs’ perceptions of using telegenetic counseling (Sjöström and Dahlgren 2002). Phenomenography is used to describe peoples’ perceptions of a certain phenomenon, using a second-order perspective (Marton 1981). Different backgrounds, geographical location and frames of reference yield a variation of experiences among individuals.

### Informants and setting

Purposeful, strategic sampling aimed to identify informants with a general knowledge of genetic counseling and a potentially wide range of different perceptions. Informants were identified through the researchers’ professional networks. This selection procedure created a broad spectrum of informants with regard to age, gender, profession, and geographic locality (Patton 2015) and included those who had experience with genetic counseling provision, either personally or through their patients. Participants were eligible if they held a position as an HCP in the southeast healthcare region in Sweden, representing one of the six health care regions in the country, with a patient uptake of approximately 1 million. This region was chosen for convenience and proximity reasons. Those that did not speak Swedish or have any experience of genetic counseling would be excluded. Eighteen informants were approached personally by one of the researchers (RP or CG) and those interested were sent an invitation email, including a participant information sheet and a consent form. The interviews were conducted either by phone or in person to accommodate the informants’ preference. The setting was the Swedish health care system, which is universal and largely funded by taxes to provide equal access for all inhabitants.

### Data collection

To enhance the trustworthiness of the findings and construct a rich picture of experiences through the contributions of a wide range of people, triangulation of informants had been performed (Shenton 2004). This involved choosing people that varied in experience, age, gender, profession and locality. An open-ended semi-structured interview guide was developed in order to make the process of interviewing comprehensive and systematic (Patton 2015). The interview guide was piloted on two clinical professionals, who were not included in the study. The final guide was adjusted to better meet the study aim. The interviews started with a short introduction from the participant (age, workplace, job title) followed by the researcher giving a brief introduction on TGC that described “a genetic counselling meeting using a video screen, allowing participants to see and hear each other in real time.” The first open-ended question allowed participant to provide their definition on genetic counseling and was used to ascertain the informant’s general knowledge on the topic, since their experiences varied. The second open question asked their perception on telegenetic counseling in clinical practice. Open-ended prompts were used to elicit thoughts about important factors, including barriers and facilitators and were asked if the informant had not already mentioned these topics. All interviews were audio-recorded and transcribed verbatim. The results were made anonymous.

### Data analysis

**Fig. 1:**
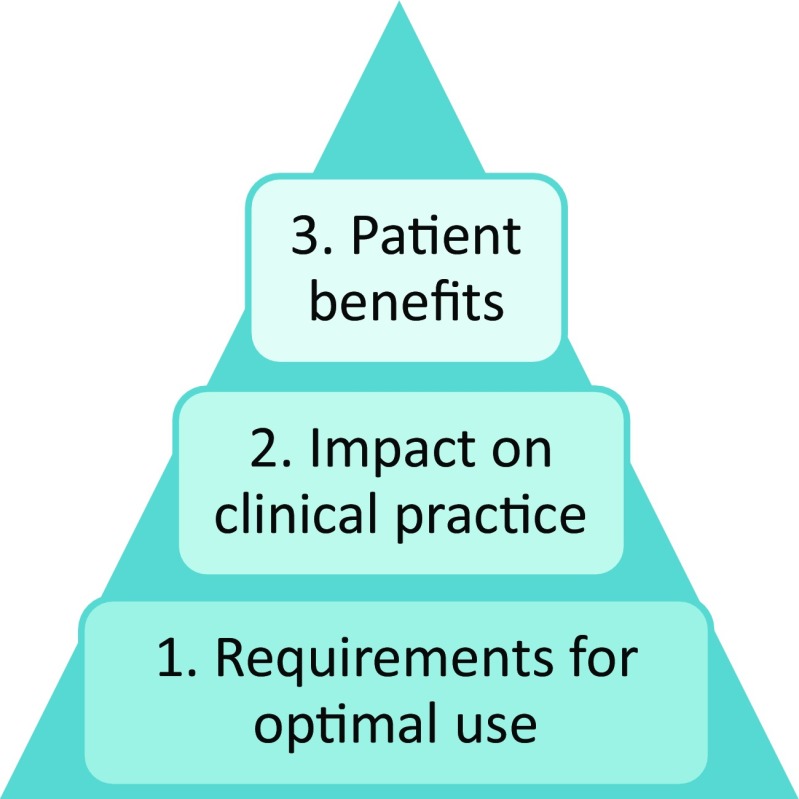
The outcome space and descriptive categories ordered hierarchically

The analysis process was performed in a step-wise manner, as described in Table 1. Significant excerpts were identified from each transcript and condensed into meaning units. From these meaning units, sub-categories emerged which were compiled into main descriptive categories. Finally, these were ordered in the outcome space (Sjöström and Dahlgren 2002), as seen in Fig. 1. The analysis was based on the researchers’ understanding from an objective perspective and was mainly performed by two researchers (RP and CG), both of whom have several years of experience working with genetic counseling patients and qualitative interviewing and some professional experience of TGC in practice, and one personal experience of a telemedicine video call. Intersubjective agreement was reached with two other researchers in the group (PJ and PN), whom have experience with clinical and implementation research. In order to establish trustworthiness, excerpts are provided in the results to show relevance for each descriptive category found in the study. The analysis procedure was based on Sjöström and Dahlgren (2002) and Marton (1981).

**Table 1:** Phenomenographic analysis procedure

Step	Procedure
Familiarization and compilation	The transcripts were read and re-read by two researchers (RP and CG) in order to obtain an overall impression of the data. Significant excerpts were marked in the text
Core components	The most significant excerpts from each participant in relation to the research questions were gathered and units of meaning identified
Grouping	The units were further classified into common sub-categories for the whole group
Comparison of categories	The sub-categories were compared to identify similarities and differences
Common understanding	As an intersubjective comparison, two other researchers (PJ and PN) provided comments on the suggested categories. This was done several times until a common understanding and agreement on the categories among the research group was achieved
Contrastive comparison	The final descriptive categories were clearly separated and assigned a hierarchical position in an outcome space, and a model was constructed to show their relationships to each other

### Ethics

Ethical approval was obtained from Regional Ethical Review Board in Linköping, Sweden, on December 14, 2016 (dnr 2016/475–31).

## Results

Out of the 18 approached participants, 16 successfully completed the interviews, which lasted between 30 min and 1 h. Interviews took place between February and May 2017, at the informant’s location of choice, 14 interviews by phone and two interviews in person, as shown in Table 2. One person declined participation due to lack of time and one interview was spoiled due to technical issues. It is assumed that none of the participants had ever used TGC in practice; however, several mentioned using video-conferencing in meetings with colleagues or family.

**Table 2:** Characteristics of informants

Informants	*n* = 16
Women	11
Men	5
Age (years), median (range)	54 (34–74)
Profession	
Doctor	8
Specialist nurse	4
Genetic counselor	2
Other	2
Interview type	
Telephone	12
Face to face	4

Clinical workplaces (number of informants): pediatrics (3), cardiology (3), clinical genetics (3), oncology (2), pediatric cardiology, rare odontological disease, obstetrics and gynecology, rehabilitation and oncogenetics

Three categories emerged from the data: (1) requirements for optimal use, (2) impact on clinical practice and (3) patient benefits. Each category consists of two or three sub-categories, in total seven sub-categories, as shown in Table 3.

**Table 3:** An overview of the units of meaning (right column) that led to the sub-categories (middle column), which together comprise the categories of description (left column) shown also in Fig. 1

Categories of description	Sub-categories	Units of meaning
Requirements for optimal use	Structural and practical issues	Joint agenda and instructions
		National harmonization
		Technical information and support, including visuals, guidelines, and web booking. Easy solutions necessary
		Guidelines regarding individual patient assessments for inclusion
		Not suitable for all patients or HCPs
	Resources needed	Patient safety and confidentiality
		Enthusiastic and willing HCPs
		Competent, experienced HCPs Provision of training for HCPs
		Patient must be well prepared
		Preparation of premises (the room, video access, on-site personnel, etc.)
	Evidence of effectiveness	Necessary to convince HCPs that this works
		No difference compared with face to face
		Evaluations to create and provide proof
Impact on clinical practice	Changes the patient meeting	Not as good as face-to-face consultations Miss out on feelings and body language Not possible to give the same support
		Clinical examination not possible
		Better than no consultation/telephone consultation
		Patients do not mind traveling
		Better consultations
	Changes to the work situation	Fear of more cumbersome administration
		Feeling insecure due to new work situation, new technical solutions, new scenarios, new assessments
		Increased flexibility for HCPs
		Easier information transfer; increased professional development
		Ambivalence
Patient benefits	Patient autonomy	Increases possibilities for and uptake of genetic counseling
		Demand from (young) patients
		Increases sense of security in certain patient groups
		Increases patient satisfaction
	Accessibility	Increases flexibility, i.e., ameliorates family clinic, consultations at home or at work
		Decreases accessibility for older generations Specialists make better assessments when working at home clinic (with access to correct information, charts, lab results, etc.)
		Increases accessibility to HCPs
		Better option than telephone consultation or no visit at all
		Time saving (faster answers, shorter visits, more than one patient at a time, no need to travel or take time off work)

### Requirements for optimal use

This category comprised three sub-categories: (a) structural and practical issues, (b) resources needed and (c) evidence of effectiveness. This main category collates factors that the informants believe are fundamental for TGC to work, such as measures for patient safety and appropriate training and guidelines for HCPs.

The first sub-category (*structural and practical issues*) addressed the importance of shared solutions on a national level, such as a simple technique and available support, confidentiality, guidelines for individual assessments, and clear instructions for providing TGC, as shown in the following quote:

…it really needs to be a simple solution that works; robust, easy to use, with as little problems as possible that us on the floor can use. (7)

The second sub-category (*resources needed*) encompassed different types of resources, including human, financial, technical and environmental. These resources were considered necessary for successful implementation and use of TGC. Several examples of resources were given, such as experienced and enthusiastic personnel, educated patients, the right equipment and location, some of what is illustrated by the following quotes:

…but like everything new it requires being introduced with a driving spirit behind it, otherwise usually nothing happens… (4)

It should be simple, no more difficult than a phone call and that is very important. Then there needs to be a certain broadband quality, so to speak, it cannot be bad quality. (11)

The final sub-category was the need for evidence of effectiveness*.* This included research and performing evaluations in order for HCPs to be convinced of the usefulness of TGC, as one informant stated:

…health care professionals prefer evidence from some study and it is difficult before the studies are done to kind of prove that this is any good. (1)

### Impact on clinical practice

The second category gathered the anticipated changes in everyday clinical practice, for both patients and the HCPs. It included perceived consequences that TGC might have on daily clinical practice. This category captured some ambivalence, as many HCPs expressed both barriers and facilitators. The category consisted of two sub-categories: (a) patient meeting and (b) work situation. It became evident when analyzing the first sub-category that most HCPs feared losing something essential in the meeting with the patient, when it would no longer be conducted face to face, as shown by the following quote:

…in the cases when supportive or empathic intervention is required, then it [TGC] can be a barrier. (13)

On the other hand, some informants believed that a meeting via video was better than a telephone call, or no meeting at all as shown below:

…to be able to see each other, see facial expressions and so on, that is an added value compared with a telephone call. (9)

…it is better to have such a meeting [TGC] than no meeting at all. (11)

In the second sub-category, changes in the clinical work situation emerged; informants disclosed some discomfort about the novel work situation, because of the need for new techniques and new clinical assessments:

…if the technical aspects do not work, it distracts everyone and that is pointless; then it is better with a telephone conversation. (9)

And at the same time being positive to effectiveness in work and travel time:

…you save travel time to satellite clinics, that is also good for the environment. (3)

However, several informants also pointed out how TGC could lead to benefits in the work situation, such as increased flexibility and improved information transfer between professionals, as illustrated by the following informants:

You can co-ordinate the expertise from around the country. (16)

…it provides possibilities for those problematic cases that we have not solved, for a few of us to collaborate in different places and go through it together… (7)

### Patient benefits

The third category comprised patient advantages that may accrue from TGC, for example, patient autonomy and increased accessibility. The first sub-category (*patient autonomy*) included increased independence, empowerment and flexibility for patients. The informants believed that younger patients requested options, such as TGC, from their health care providers and it could be easier from people with psychiatric illness to receive TGC than in-person genetic counseling. It was believed to increase the patient’s level of overall satisfaction, as illustrated by this quote:

…it can be a matter of choice and for some people it can be a relief to sit in their normal environment and ask these questions… it can provide increased integrity for some. (13)

Overall accessibility to genetic counseling was thought to improve with TGC, despite long distances, time constraints, or family members in various locations, as illustrated by the following quotes:

They [patients and family] can live in different parts of the country and still all meet and get the same information… (15)

…and the need to see a genetic counsellor is great and resources scarce, I know, so this is a superb way of reaching specialist knowledge wherever you live. (1)

However, some informants thought that TGC might disadvantage older patients, those without much technical knowledge and experience, as shown by this response:

…but if they [elderly people] get help with the technical parts, they can go to their local health care provider to get help with the technology, then I do not believe it will be a big problem. (9)

## Discussion

This study aimed to identify factors that influence the implementation and use of TGC in clinical practice, according to HCPs in Southeast Sweden. Three main categories emerged from the analysis: (1) perceived requirements for optimal use, (2) impact on clinical practice and (3) patient benefits. The categories consist of different determinants that can be important factors for implementation, acting as barriers and/or facilitators for the use of TGC.

The most fundamental requirements to facilitate the use and implementation of TGC among HCPs seem to be adequate resources to enable optimal use, as well as evidence of the effectiveness and satisfaction with TGC. These requirements are in line with findings from implementation research, underscoring that diffusion alone is not sufficient enough to achieve uptake and adoption of innovations in health care settings (Grol et al. 2005). A study on TGC from the Netherlands showed that 24% of HCPs in genetics had access to appropriate video-conferencing equipment to provide TGC, but only a third of them use it for patient counseling (Otten et al. 2016a). These numbers suggest that TGC is underutilized, potentially because specific requirements for optimal use, as identified here, have not been taken into consideration, or been recognized until now.

The resources that are required by the HCPs are both human and technological in nature. Positive attitudes among HCPs and individuals and a driving spirit in the clinic are important for successful introduction and use. This finding is consistent with the Diffusion of Innovations theory by Rogers (2000), which highlights the importance of intermediary actors (opinion leaders, change agents, and gatekeepers) for successful adoption and implementation. On the other hand, dependence on a single person may jeopardize implementation, making it too dependent on a sole person.

In view of the technological prerequisites mentioned by informants such as bandwidth, connection quality and familiarity using the internet, access and digital literacy in Sweden ranked third on the EU digital economy and society index in 2017. Ninety-one percent of the population have access to the internet, 75% have basic digital knowledge, and 51% already use video calls (European Comission 2017). These figures point to favorable conditions for the implementation and use of TGC, at least in Sweden.

The informants’ also perceived that TGC can have an impact on current clinical practice concerning the work situation and patient meeting. Informants anticipated reduced travel time to attend distant clinics for both patients and themselves, as well as improved access and uptake for patients who otherwise would not attend the clinic. Some informants believed that TGC could make patients who experience social or hospital anxiety feel more at ease and thus more likely to participate in a consultation. This is in line with previous studies on the impact of TGC that have shown savings of both time and money as well as increased perceived personal control (Zilliacus et al. 2009).

Several studies have measured patient and provider overall satisfaction and effectiveness of TGC, and found them to be nearly equal to in-person consultations (Zilliacus et al. 2009; Zilliacus et al. 2010; Meropol et al. 2011; Hilgart et al. 2012; Buchanan et al. 2016; Otten et al. 2016b, c; Weissman et al. 2017; Voils et al. 2017). However, many informants in our study emphasized the importance that patients can choose their preferred counseling mode, be it via video or in person. This finding is consistent with the ideals of person-centered care, allowing the patient to choose, emphasizing a holistic and biopsychosocial patient approach (Olsson et al. 2013). Moreover, informants anticipated improved collaboration between professionals involved with the same patient, also adding to patient-centered care.

Not surprisingly, there were some anticipated barriers. For example, technical solution problems and increased administration raised concerns that TGC might require more time and effort, instead of the opposite. A review from 2012 suggests that this type of resistance against adopting TGC is reduced when provided with appropriate training, growing experience and confidence (Hilgart et al. 2012), aspects also found in this study. For example, education for HCPs regarding the use of the technology was considered pivotal. According to the HCPs, a major drawback with TGC is the loss of physical closeness to the patient. Physical closeness is considered by many an important dimension to the patient meeting. This finding is in line with other studies on TGC, which have reported loss of non-verbal ques and inhibition of rapport. However, informants are not specific on what is missing in TGC compared with in-person consultations and previous findings show that patient satisfaction tends to be higher than HCP satisfaction (Hilgart et al. 2012). Perhaps this has to do with differences in desired outcomes from a consultation for patients and HCPs, or any other factors not investigated here.

HCPs did believe that TGC could benefit their patients in different ways. It provides an opportunity to reach a wider patient group, for example, patients who live far away from health care centers, have many family members, have psychiatric illnesses, or patients who have difficulty taking time off work. TGC is also perceived to meet the expectations of younger patients. In summary, TGC can reach patients who otherwise would have telephone counseling or no counseling at all, a group that is expected to grow with more mainstreaming in the era of genomics. Patients’ ability to choose their preferred delivery mode (i.e., TGC or in person) is deemed important to avoid exclusion of any specific patient groups, e.g., elderly or sick people. These findings are in agreement with the results of several other studies (d'Agincourt-Canning et al. 2008; Baumanis et al. 2009; Buchanan et al. 2016; Weissman et al. 2017).

From a broader perspective, it is possible to interpret the three different categories found in this study in a hierarchical order, corresponding to important factors that influence the implementation and use of TGC. This is illustrated in the outcome space in Fig. 1: (a) requirements for optimal use (*bottom*) need to be met in order to achieve, (b) an impact on clinical practice (*middle*), which then allows for (c) patient benefits (*top*). The figure demonstrates the order in which the factors need to be addressed, from bottom to top, to achieve successful implementation of TGC and to realize the patient benefits.

Our findings show that the informants perceive both drawbacks and benefits with TGC. These results suggest considerable ambivalence on the use of TGC in clinical practice, because it alters ways of working and thus is a potential threat to some professionals’ accrued clinical competences. Ambivalence emerges in all three levels in Fig. 1. Most informants say that TGC is something beneficial that needs to happen fairly soon, at the same time pointing out that “something” seemingly qualitative will be lost in the patient meeting, be it body language, pheromones, physical closeness, or disruptions due to technical disturbances. This can be interpreted as an expression of attitudinal ambivalence, a concept introduced by Scott (1969), who defines it as a psychological state in which “a person holds mixed feelings (positive and negative) towards some psychological object” (Gardner 1987). Research shows that higher levels of attitudinal ambivalence create discomfort, which leads people to seek out consensus information in order to solve the conflict and reduce dissonance (Zemborain and Venkataramani Johar 2007). Research in the field, including this study, can provide such additional information for HCPs and decision makers. The low uptake despite availability of TGC shown previously (Otten et al. 2016a; Vrecar et al. 2017) is possibly due to important factors being overlooked in the implementation process. Our findings may shed light on how TGC can be successfully implemented and more widely used within clinical genetics, as compared to other specialties. Naturally, when introducing a new way of working there is resistance and a sense of loss of control. In this study, most informants agree, similarly to Rushmer (2004), that these hurdles can be overcome with the right support, educational interventions, and detailed information for both HCPs and patients. Rushmer observed many factors that contribute to individuals resisting change to current practice, including established and efficient habits, experiencing security, and fear of the unknown. These factors point to the importance of preparation and groundwork before introducing new ways of working (Rushmer 2004). The findings from our study are potential factors to consider before implementing TGC, as they reflect what a select group of HCPs require to use TGC in clinical practice.

### Strengths and limitations

This study has some limitations that should be considered when interpreting the findings. Firstly, all results are dependent on the informants’ and researchers’ experiences and on the context in which this research took place. Sixteen interviews may seem a small sample. However, since the field of practice is relatively small in Sweden, the sample size was deemed sufficient because there are a limited number of suitable informants with relevant experience. This number of interviews is generally considered sufficient to obtain informative findings in phenomenography (Larsson and Holmström 2007). The transferability of the study findings might have improved if HCPs from all over Sweden had been included, including more genetic counselors, unfortunately due to time and cost restrictions that was beyond the scope of the current study. To enhance the credibility of the findings, triangulation of informants was performed. The main researcher (RP) has extensive experience in providing genetic counseling, as well as knowledge in analyzing interview responses. Of course, this may affect the objectivity, because it is not possible to be without preconceived notions. Therefore, responses were cross-compared by other researchers in order to increase trustworthiness and obtain objectivity (Shenton 2004). The sub-categories were intended to be completely separate; however, this was difficult to achieve, to some part because of the ambivalence found in the HCP’s answers. In addition, an inherent problem in this research was asking individuals to think about a concept that they have not experienced themselves, because TGC does not yet exist in our clinical practice. It is therefore important to be cautious in generalizing results from an exploratory, qualitative study that has taken place in a specific setting.

## Conclusion and implications

In summary, participating HCPs identified three factors necessary for implementation of TGC in clinical practice. There were the requirements for optimal use, the impact on clinical practice and the patient benefits. These factors could be considered in order to improve access and uptake of TGC in Sweden and in other countries with similar health care systems, especially considering mainstreaming of genetics and in the era of genomic medicine. Interestingly, the present study found that participating HCPs show a great deal of ambivalence towards TGC, which may be important to explore further in order to deduce the factors that are most important to achieve successful implementation. In summary, TGC could benefit not only remote patient populations, as described in previous research, but perhaps also meet the increasing demand in the wake of the genomics era. Thus, perceptions of more HCPs as well as patients should be sought in future exploratory research.
